# A case report of an ischaemic stroke, caused by a primary cardiac intimal sarcoma

**DOI:** 10.1186/s12872-023-03090-5

**Published:** 2023-02-01

**Authors:** Joshua Lloyd, Navinee Gilliat, Graeme Porter, Francesco Pirone

**Affiliations:** 1 Te Whatu Ora Health New Zealand – Bay of Plenty, 829 Cameron Road, Tauranga South, Tauranga, 3112 New Zealand; 2 Te Whatu Ora Health New Zealand – Waikato, 183 Pembroke Street, Hamilton, 3204 New Zealand

**Keywords:** Stroke, Intimal sarcoma, Cardiology, Case report, Primary cardiac malignancy

## Abstract

**Background:**

Intimal sarcomas are an extremely rare type of primary cardiac malignancy. They most commonly present with symptoms of cardiac dysfunction. We present a case of intimal sarcoma identified without any cardiac signs or symptoms. Cardiac sarcomas historically carry a very poor prognosis.

**Presentation:**

A 57-year-old man presented with a sudden onset of left limb weakness and disorientation. MRI brain identified an acute ischaemic stroke in the right anterior temporal lobe. Four months later, he presented again with transient left arm weakness. The patient had a normal cardiovascular examination and ECG. All other initial investigations for cryptogenic stroke were non-contributory. The patient did not initially get an echocardiogram. When this investigation was performed, after his second presentation, a large pedunculated mass was present in his left atrium. This was resected and identified histologically as a primary intimal sarcoma of his left atrium. The patient was treated with post-operative radiotherapy but declined chemotherapy. He recovered well post-operatively but subsequently passed away 14 months after diagnosis.

**Conclusions:**

It is possible for primary cardiac malignancies to present with only symptoms of systemic emboli. For this reason, echocardiography is a crucial investigation in cases of cryptogenic stroke. Some stroke guidelines do not definitively support routine echocardiography. Primary intimal cardiac sarcoma is a very rare condition with a poor prognosis. The literature is limited to case reports and optimal management is with surgical resection where possible. The role of post operative radiotherapy and chemotherapy is uncertain.

## Background

Even as a broader group, primary cardiac malignancies are rare, with 34 cases per 100 million persons. Cardiac sarcomas make up approximately 65% of primary cardiac malignancies [1]. Intimal sarcomas appear to be the rarest subtype of sarcoma, with only a small number ever reported in the literature [2, 3].

Most commonly this malignancy presents with chest pain, oedema, dyspnoea and palpitations [1, 4]. We report a case where the patient presented with purely neurological symptoms, with no clinical clue of his underlying cardiac malignancy. We are aware of only one other case of primary cardiac sarcoma presenting with stroke. In that case it is unclear if there were abnormalities on ECG or cardiovascular examination [2].

Histologically, intimal sarcoma is identified by poorly differentiated spindle shaped cells that may resemble smooth muscle. These tumours can be difficult to diagnose both histologically and radiologically. This, along with their rarity, mean there are frequently delays in diagnosis [5]. The incidence of cardiac sarcoma is increasing, so clinicians may be faced with this condition in the future [1].

Most previous cases have been treated with surgical resection, where possible. There is a possible role for radiotherapy or systemic chemotherapy. Management can be very difficult, with very few previous cases to judge whether radiotherapy or chemotherapy have any benefit. The prognosis of this condition is extremely poor, with mean survival quoted as being between 3 and 12 months [2].

## Case presentation

### Presentation

A fit 57-year-old building inspector presented, by ambulance, with sudden onset left limb weakness and “shaking”, associated with confusion. The symptoms lasted minutes and had resolved before arrival to the Emergency Department. He had a background of homozygous ZZ phenotype alpha-1-antitrypsin deficiency with associated mild bi-basal bronchiectasis and a distant background of a resected melanoma.

Four months later, he presented again with a sudden onset of episodes of left arm weakness. The episodes lasted several minutes, with complete resolution in between, over a period of eight hours. He had no other neurological, cardio-respiratory, or systemic symptoms of note. His neurological and cardio-respiratory examination was normal.

### Investigations

The patient’s most recent imaging prior to this presentation was a CT chest from 3 years earlier, which showed mid to lower zone panlobular emphysematous change with associated bronchiectasis and no obvious cardiac abnormality.

**Fig. 1:**
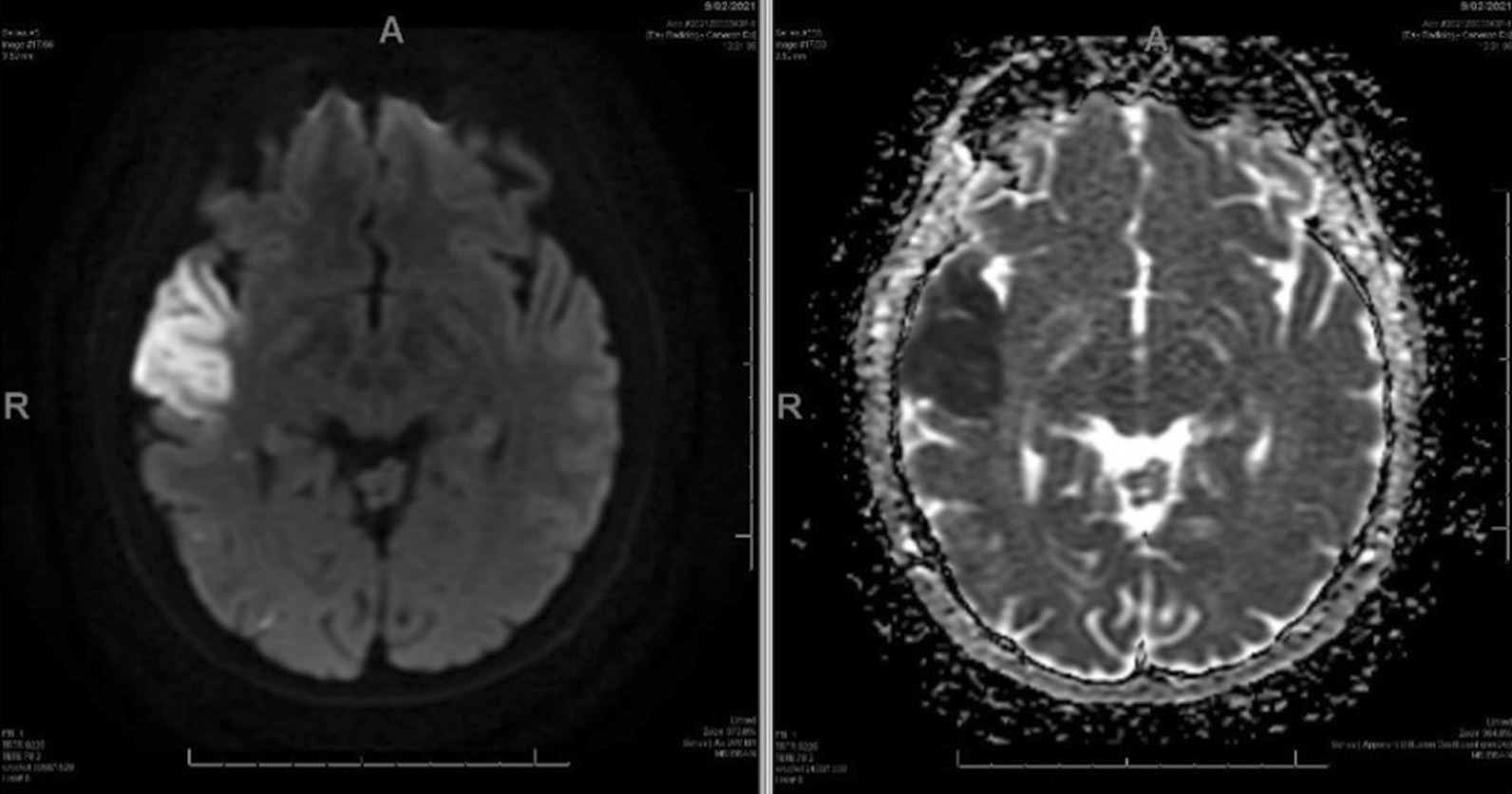
MRI brain at initial presentation

At the initial presentation the patient had unremarkable basic bloods. Baseline ECG and 72 h of continuous ECG monitoring demonstrated no atrial fibrillation or other arrhythmia. CT and CTA of head and neck showed normal appearance of intra and extracranial arteries with no significant atherosclerotic disease. MRI brain revealed acute right anterior temporal lobe infarction (Fig. 1).

Given the higher incidence of ANCA associated vasculitis in patients with alpha 1 antitrypsin deficiency, a vasculitis screening panel was sent. This was still pending at the time of discharge, but eventually returned as negative.

**Fig. 2:**
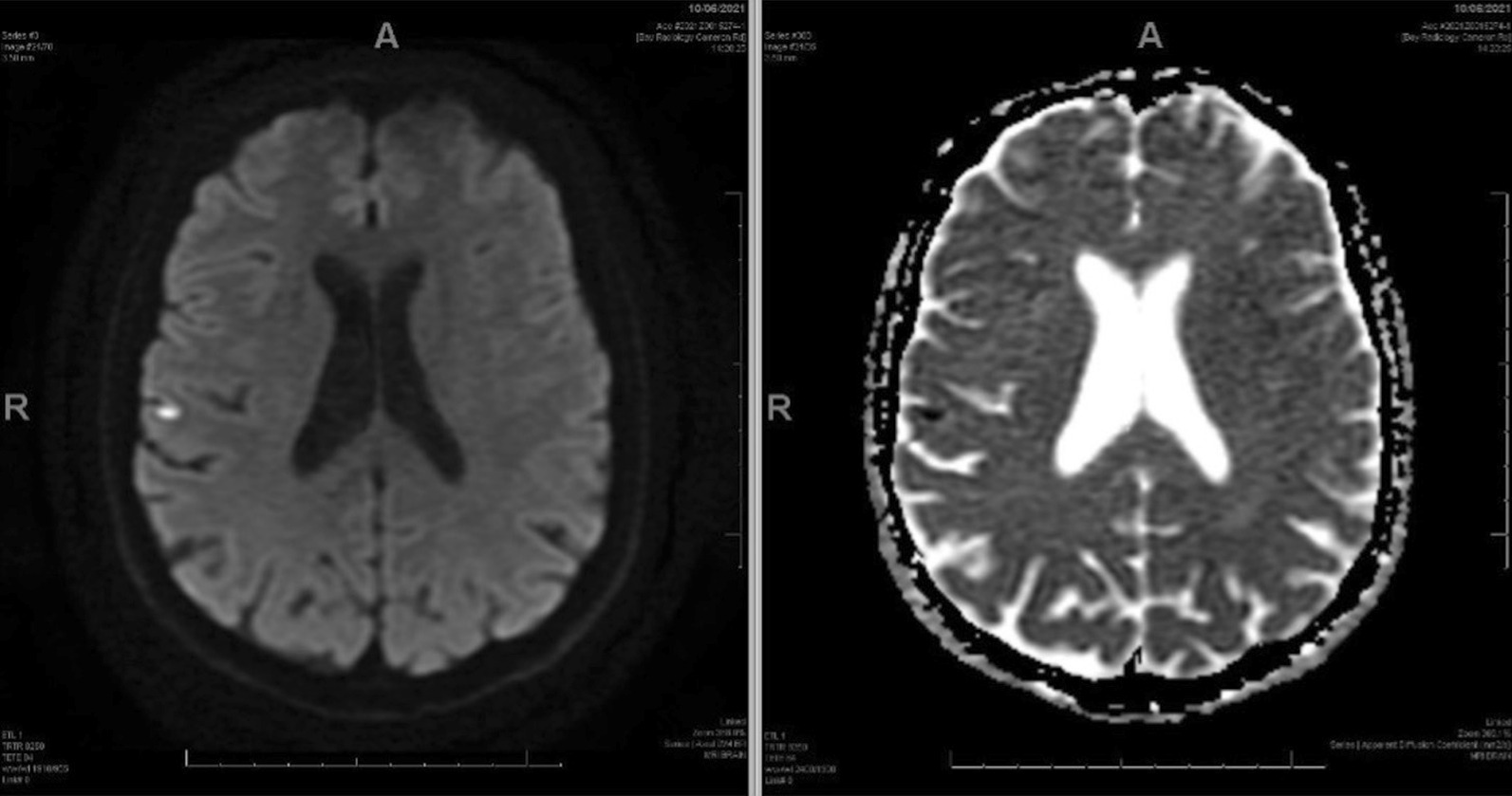
MRI brain at the second presentation

When the patient re-presented with further episodes of left sided weakness, repeat MRI brain showed a small focus of acute restricted diffusion at the left superior temporal gyrus and gliosis at the site of the previous temporal infarct (Fig. 2).

Repeat CTA of head and neck, telemetry and vasculitis screen did not reveal any clear cause for ischaemic stroke. His ECG showed normal sinus rhythm.

**Fig. 3:**
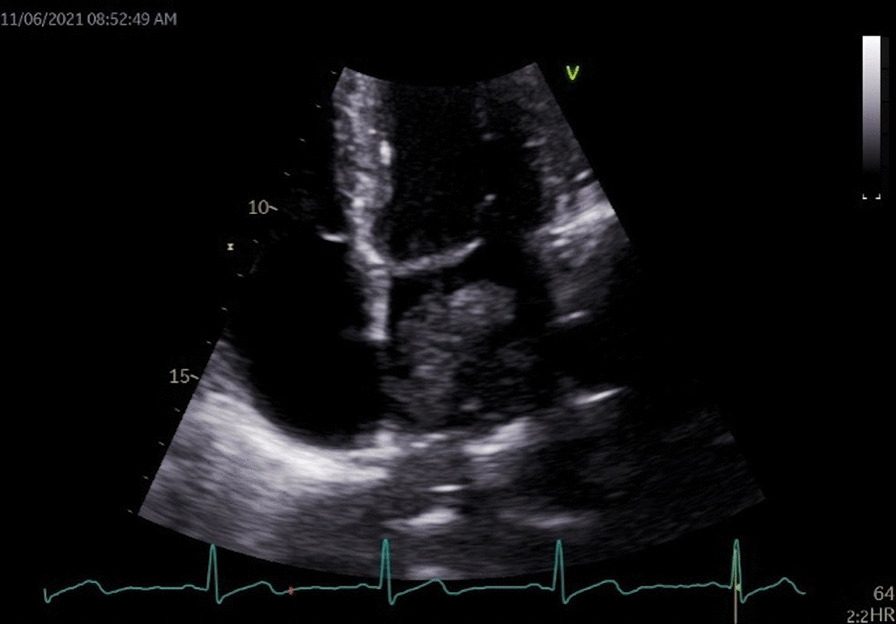
Pedunculated mass within the left atrium

Echocardiography was not performed at the patient’s first presentation, based on our interpretation of local guidelines [6, 7]. This is explained further in the discussion. When echocardiography was performed at the patient’s second presentation, it revealed a very large pedunculated mobile mass approximately 65 × 35 mm which filled the left atrium and prolapsed through the mitral valve (Fig. 3).

CT chest/abdomen/pelvis subsequently showed a left atrial lesion with extension into bilateral inferior pulmonary veins and left ventricle and several enlarged retro-cardiac lymph nodes. There was no evidence of any other lesion.

### Treatment

**Fig. 4:**
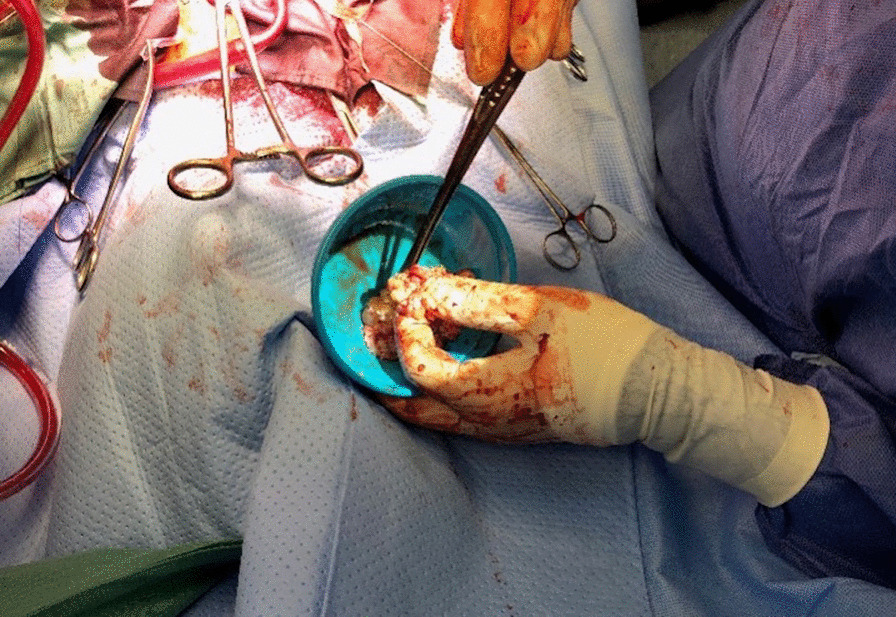
Intra-operative image of the atrial sarcoma, immediately after resection

The patient underwent a left atrial mass excision on the third day of his second admission. (Fig. 4).

**Fig. 5:**
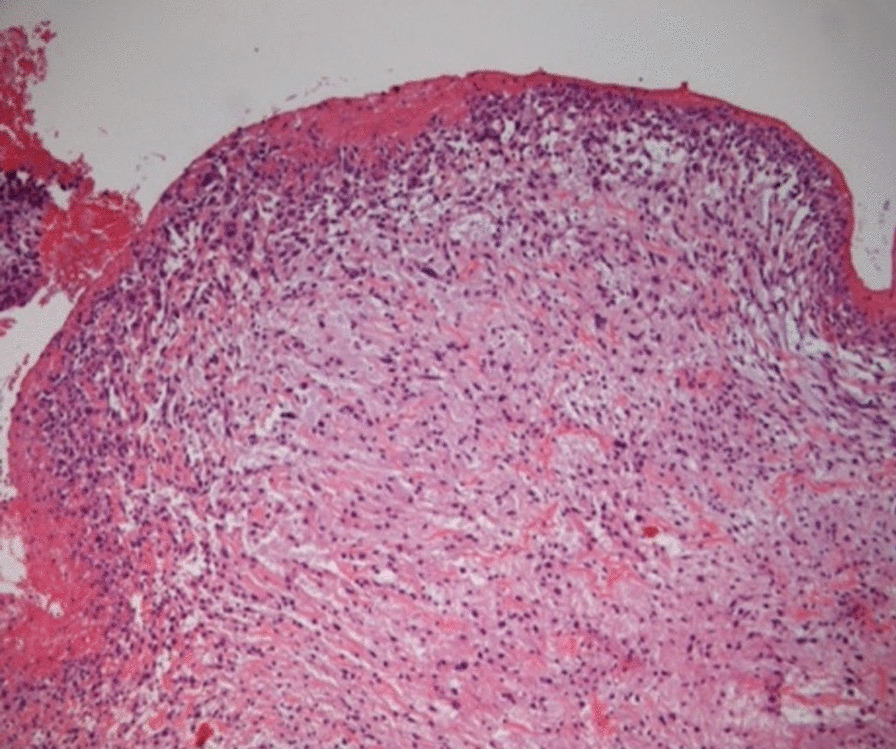
Hematoxylin and eosin staining of a section of the excised mass, at 40 × magnification. Pleomorphic spindle cells within a myxoid background are seen below the eroded endothelial layer, accompanied by a surface fibrinous exudate

The histology was reported as 90 mm pleomorphic spindle cell tumour. (Fig. 5) Tumour was seen to the inked margin.

He recovered well. He was discharged on the thirteenth post-operative day.

After discussion at the national sarcoma multi-disciplinary meeting, this was felt to be an intimal sarcoma. The patient underwent outpatient post-operative radiotherapy to the sarcoma site. He received 3 months of warfarin and aspirin post operatively and then moved to aspirin monotherapy.

### Outcome and follow up

The patient recovered well from this treatment. He returned to work, with a Modified Rankin Score of 0. Three-monthly echocardiographs and CT chest, abdomen and pelvis did not show any evidence of disease recurrence. Despite these reassuring investigations, the patient presented acutely to the emergency department 14 months after his diagnosis with symptoms of a small bowel obstruction. CT of his abdomen identified probable metastatic disease. He passed away several days later in hospital.

## Discussion

Very few cases of primary intimal sarcoma have been previously reported. While previous cases have tended to present with signs of cardiac dysfunction [1, 8], this case did not have any signs to indicate his underlying cardiac malignancy.

Other, more common forms of left atrial tumours such as left atrial myxoma are recognized as “high risk” for embolization by consensus [9]. There are reports of other subtypes of cardiac sarcoma presenting with stroke [4, 10]. It is therefore reasonable to consider that our patient’s neurological symptoms were embolic.

This patient had a normal cardiovascular history and examination, as well as normal ECGs and cardiac monitoring. He had no known risk factors for cardiovascular disease. Despite this, he had an underlying primary cardiac malignancy.

Echocardiography was crucial in establishing this diagnosis. Current Royal College of Physicians stroke guidelines recognize the importance of echocardiography in stroke, however in the “Choosing Wisely” section, they say to NOT perform echocardiograms routinely. They suggest selecting patients with history of structural cardiac disease or abnormal physical or ECG findings [6].Our case did not meet these criteria. The Australasian stroke guidelines, adopted by our institute, suggest echocardiography considering “based on an individual patient factors” [7]. While you could make an argument that these guidelines support echocardiography, they are not completely clear.

Subsequent to this case, we strongly advocate for performing echocardiography as a first line investigation in cryptogenic stroke, even in the absence of any cardiac signs. This may lead to earlier identification and more successful treatment of these rare cardiac malignancies. We suggest that local clinicians follow guidelines released by the Cardiac Society of Australian and New Zealand. They are more definite with their recommendation for echocardiography. They suggest transthoracic echocardiography for all cryptogenic stroke in people under 60 years-old [8].

Cardiac sarcomas generally carry a very poor prognosis. One year survival is only 47%. Three-year survival is 21% [1]. Intimal sarcomas as a subtype appear to have even poorer outcomes, with mean survival between 3 and 12 months [2]. Treatment with surgical resection and post operative radiotherapy, in our case, led to the patient having a period of good quality of life and no macroscopic evidence of disease. Sadly, he later died due to disease recurrence in the abdomen, 14 months after diagnosis and resection. It is difficult to establish optimal treatment in this disease given its high malignant potential and rarity.

Despite their extreme rarity, the incidence of cardiac sarcomas is increasing. [1] It is possible clinicians may come across them in the future.

### Learning points

- In cases of cryptogenic stroke, even in the absence of cardiac symptoms and signs, trans-thoracic echo should be considered.
- Cardiac malignant tumours most commonly present with chest pain, dyspnoea and decompensated heart failure. They can present with symptoms of systemic emboli only, with no cardiac history or physical signs.
- The management of primary cardiac malignancy is complex and involves a large range of specialist services. Surgical resection, where possible, is the mainstay of treatment.
- In general, the prognosis of a primary intimal cardiac sarcoma is poor, based on a small number of case studies.

## Data Availability

Data sharing is not applicable to this article as no datasets were generated or analysed during the current study.
